# Hearing aid fitting at SUS (Brazilian Public Health Care System) compared with a compact fitting model

**DOI:** 10.5935/1808-8694.20130051

**Published:** 2015-10-04

**Authors:** Maria Cecília Bevilacqua, Orozimbo Alves Costa Filho, Eliane Aparecida Techi Castiquini, Ticiana Cristina de Freitas Zambonatto, Marina Morettin, Adriane Lima Mortari Moret, Regina Célia Bortoleto Amantini

**Affiliations:** a Full Professor - University of São Paulo - Bauru Campus; Professor - Department of Speech and Hearing Therapy at FOB/USP; Coordinator of the Audiological Research Center - CPA- HRAC/USP, Bauru Campus.; b Full Professor - University of São Paulo, Bauru Campus. Professor - Department of Speech and Hearing Therapy at FOB/USP; Coordinator of the Audiological Research Center - CPA - HRAC/USP, Bauru Campus.; c MSc. in Communication Disorders; Speech and Hearing Therapist - Hearing Health Division - Craniofacial Anomalies Rehabilitation Hospital (HRAC/USP).; d MSc in Rehabilitation Sciences; Speech and Hearing Therapist - Hearing Health Division - Craniofacial Anomalies Rehabilitation Hospital (HRAC/USP).; e MSc in Public Health; Speech and Hearing Therapist; Laboratory Specialist - Speech and Hearing Department - School of Dentistry - FOB/USP; Researcher - Center of Audiological Research - CPA/USP- Bauru.; f PhD in Human Communication Disorders; Professor at the Department of Speech and Hearing Therapy - FOB/USP; Vice-Coordinator at the Center of Audiological Research - CPA - HRAC/USP - Bauru Campus.; g PhD in Human Communication Disorders; Administrative Director - Hearing Health Division - Craniofacial Anomalies Rehabilitation Hospital (HRAC/USP). Department of Speech and Hearing Therapy - Dentistry School of Bauru. Audiological Research Center - Craniofacial anomalies Rehabilitation Hospital (HRAC/USP). University of São Paulo, Bauru Campus.

**Keywords:** adult, hearing, hearing aids, hearing loss, outcome and process assessment (health care)

## Abstract

In the present days it is critical to identify the factors that contribute to the quality of the audiologic care provided. The hearing aid fitting model proposed by the Brazilian Unified Health System (SUS) implies multidisciplinary care. This leads to some relevant and current questions.

**Objective:**

To evaluate and compare the results of the hearing aid fitting model proposed by the SUS with a more compact and streamlined care.

**Method:**

We conducted a prospective longitudinal study with 174 participants randomly assigned to two groups: SUS group and Streamline group. For both groups we assessed key areas related to hearing aid fitting through the International Outcome Inventory for Hearing Aids (IOI-HA) questionnaire, in addition to evaluating the results of Speech Recognition Index (SRI) 3 and 9 months after fitting.

**Results:**

Both groups had the same improvement related to the speech recognition after nine months of AASI use, and the IOI-HA didn't show any statically significant difference on three and nine months.

**Conclusion:**

The two strategies of care did not differ, from the clinical point of view, as regards the hearing aid fitting results obtained upon the evaluation of patients in the short and medium term, thus changes in the current model of care should be considered.

## INTRODUCTION

Since 1988, Brazil has been establishing a dynamic and complex public health care system (*Sistema Único de Saúde* - SUS), based on the principles of health as a right of the citizen and a duty of the State. SUS aims at providing broad and universal preventive and curative healthcare, by managing and providing decentralized healthcare services, promoting community participation in all levels of public administration^1^. Since it is considered an internationally unique model, it must be constantly assessed.

Today, to produce scientific evidence and use it in clinical practice and in the management of healthcare services are paramount to qualify the work of healthcare teams and the management which stems from practical experience and formal research^2^. Areas involving technologies require studies using such information in order to assess policies and treatment effectiveness aiming at optimizing public funds^3^, ^4^, ^5^ and provide quality care to patients.

Difficulties in the field of hearing disorders involve the bio-psycho-social aspects of a person and its incidence and consequences are more pronounced in developing countries, where there is limited supply of services, very few trained professionals and little knowledge on how to deal with such challenges. In developed countries, hearing impairment has had high financial costs, as well as in developing and underdeveloped countries^6^. The main problems faced by healthcare organizations have been the misdistribution of financial resources, inefficacy, the growing costs and inequalities in access to healthcare^7^. If resources are to be used efficiently, it is paramount to know what can be expected from rehabilitation and which are the most efficient means of rehabilitation for different groups of people with hearing impairment^8^.

In Brazil, since 2004, with the approval of the National Policy of Hearing Health Care^9^, the supply of hearing impairment treatment by the Federal Government is different from that in other countries in regards of dispensing Individual Sound Amplification Devices (ISAD), the entire treatment is covered by the public healthcare system (SUS), from prevention through treatment, and the patient incurs no expenses concerning the ISAD received, paying only for its maintenance (batteries, cleaning, repairs, etc.). Thus, public expenditures are high in regards of hearing disorders treatment in this country.

Ordinance 587^10^ proposes systematization with specialized multiprofessional care to people with hearing loss in the medium and high complexity levels. Basic care still is a goal to be reached. Therefore, the healthcare model in Brazil is internationally unique and, in its 7 years of development many issues, of different natures, have been raised.

So far, the effectiveness and efficiency of this ordinance regulating the model proposed has not been broadly analyzed. The National Plan of Rights for People with Disabilities is already launching a new ordinance for this field without having assessed the care and quality of this care provided to patients. Today, we know that it is paramount to pinpoint the factors contributing to the quality of the audiological care provided^11^, with the necessary resources to avoid wasting funds.

With this in mind, this study aimed at assessing and comparing the results of ISAD fitting in the model proposed by SUS, with a more compact and streamlined model of care.

## METHOD

### Place where the study was carried out

We carried out a longitudinal prospective study. The entire investigation was done in the Center of Audiological Research of the Hospital of Craniofacial Anomalies (CPA/ HRAC-USP) - University of São Paulo, Bauru Campus. The patients recruited were referred to Audiological Diagnosis at the Auditory Health Division of the Craniofacial Anomalies Rehabilitation Hospital (DSA/HRAC-USP) - Bauru Campus, which is a clinic listed in the high complexity roster of the Public Healthcare System - SUS.

### Ethical Remarks

The present study was approved by the Ethics in Research Committee of the associated institution (Research Protocol # 327/2008), linked to the CNPq project # 476233/2008-9. All the volunteers received an informative letter and signed the Informed Consent Form, thus consenting with the execution and disclosure of this study and its results, as per resolutions 196/96 and MS/CNS/CNEP n 196/96 of october 10, 1996.

### Sample selection

The initial sample was made up of 174 participants from both genders. The following criteria had to be met for patient enrollment:

• Age of 15 years and older
• Unilateral or bilateral sensorineural or mixed hearing loss
• With or without previous experience with an ISAD
• Current users or potential users of a behind-the-ear hearing aid (ISAD)

Individuals with air-conduction-type hearing loss and those with retrocochlear changes and those with disorders associated with the hearing loss (such as blindness, severe cognitive disorders, brain palsy, and others) were taken off the study.

The participants were randomly assigned to two groups: SUS group (seen at the Hearing Health Division following the specifications established on Ordinance 587^10^) and Streamline group (seen at the Hearing Health Division, following a more compact and streamlined model of care defined by the researchers and described below).

In this randomization we included all the patients in the care reference month and those who met the inclusion criteria established in the present study. For such, the receptionist of the Hearing Health Division assigned all the 174 unfilled charts in both groups, with their respective Informed Consent forms. The patients who did not come to the appointment or those who refused to participate in the study were taken off the sample. Thus, the next name taken from the list was invited to participate in the present study, within the aforementioned sequence.

### Procedures

For data collection, two audiologists from the Hearing Health Division were responsible for seeing the patients and they alternated between the SUS and the Streamline groups. In both groups we investigated the following sociodemographical and audiological variables: age, gender, schooling, socioeconomic status and hearing loss classification (hearing loss mean value in the speech frequency from the best ear).

Below, we list the procedures and instruments used to develop this field study.

#### SUS group

After the patient entered the clinic, their audiological diagnosis was proposed according to Ordinance SAS/MS nº 587^10^ in both groups studied. The study started after the diagnosis, when the participants were selected based on the inclusion criteria. The procedures in this group involved the proposal present in the above-mentioned Ordinance:

1. Selection of three retroauricular ISADs
2. Selection of a proper prescription method
3. Speech perception assessment with and without the three ISADs selected by means of specific procedures
4. Functional gain assessment by means of a free-field audiometry without ISAD and with the three models of hearing aids selected
5. Measurement assessment with the probe microphone (insertion gain) with the three models of hearing aids (ISADs) selected
6. Indication and fitting of ISAD, with the best results obtained in the procedures listed above
7. Instructions to the patients or guardians about use, handling and care with the fitted hearing aid, as well as the benefits and limitations of the amplification
8. Following up at three and nine months after fitting.

These eight items are prescribed by the SUS-guiding ordinance, besides consultations with other professionals, such as psychologists, social workers and ENT physicians at the hearing aid indication and fitting.

Consultation time for this group was not established.

#### Streamline model

In this model, the team was made up only by a speech and hearing therapist (audiologist) and the difference would be only related to the ISAD selection and fitting, following the same proposal during diagnosis stage. The procedures were done by only one audiologist during all the stages and in follow up. In this model, the multidisciplinary team acted only in the cases of hearing loss, those who were not part of the investigation. Moreover, the equipment used in the care of these patients was all located in one single room.

The procedures in this group involved:

1. Selection of the prescription method: NAL-NL1
2. Selection of only one retroauricular ISAD model from the prescription rule
3. Speech perception assessment with and without the single ISAD model selected, by means of specific procedures
4. Measurement assessment with a probe microphone (degree of insertion) with the single ISAD model selected
5. Instruction to the patients or guardians regarding the use, handling and care concerning the fitted hearing aid, as well as benefits and limitations of the amplification
6. Follow up at three and nine months after fitting.

The visit in this group should not take longer than 30 minutes in cases of unilateral hearing loss, and 45 minutes in cases of bilateral hearing loss, for selection and follow up.

### Instruments

At three and nine months of follow up we assessed important domains for ISAD fitting for both groups - SUS and Streamline - (use, benefit, satisfaction, participation restriction, activity restrictions, impact on others and quality of life), and we employed the International Outcome Inventory for Hearing Aids (IOI-HA) questionnaire, proposed by Cox et al.^12^ and translated by Bevilacqua et al.^13^, besides assessing the results of the Speech Recognition Index (SRI).

#### Administration of the International Outcome Inventory for Hearing Aids (IOI-HA) questionnaire

The IOI-HA was administered after 3 and 9 months of hearing aid use.

The IOI-HA questionnaire analysis was carried out considering the score from each question and the total score obtained by adding the scores from the seven questions. Considering the seven questions, the alternatives correspond to a minimum score of one (1) and maximum of five (5), from left to right. The total score is given by the summation of the points obtained from each one of the seven questions. Thus, the minimum total score was 7, and the maximum was 35 points. A higher score indicated better results in relation to the ISAD fitting.

### Analysis of Results

The descriptive analysis of the results was carried out by means of the Stata software, version 10.0. The Chi-square and Mann-Whitney tests were utilized to check whether there were differences between the groups as far as gender is concerned, socioeconomic class, schooling, age, ISO mean of the audiometric thresholds (500, 1 kHz, 2 kHz and 4 kHz) of the middle ear and Speech Recognition Index (SRI).

The Mann-Whitney test was utilized to compare the IOI-HI scores between the groups. The Wilcoxon test was utilized to compare the IOI-HA scores between the three and nine months of ISAD use for both groups.

The Wilcoxon test was utilized to check whether there was a difference between the Speech Recognition Index (SRI) in the two models investigated before fitting the hearing aid, and 3 and 9 months afterwards. The differences in SRI results between the two groups were investigated by the Mann-Whitney test.

For all the cases we adopted a 5% level of significance.

## RESULTS

The results from the sociodemographical variables analysis [age range, gender (Table 1), schooling and socioeconomic status (Table 2) and audiological procedures (Table 3)] showed that the distribution of these characteristics between the two groups was homogeneous.

**Table 1:** Demographic data of the 174 patients, 92 in the SUS Model and 82 in the Streamline.

	SUS		Streamline	
	n	%	n	%
Age range				

15a1m-30a	14	15.22	15	18.29
30a1m-45a	13	14.13	12	14.63
45a1m-65a	63	68.48	55	67.07
65 years or older	2	2.17	0	0.00
Total	92	100.00	82	100.00

Gender				

Females	51	55.43	47	57.32
Males	41	44.57	35	42.68
Total	92	100.00	82	100.00

Value of *p*: Age range: *p* = 0.195; Gender: *p* = 0.802.

**Table 2:** Distribution of the 174 patients, 92 in the SUS and 82 in the Streamline Model, in regards of schooling and socioeconomic status.

	SUS		Streamline	
	n	%	n	%
Schooling				

Illiterate	30	32.61	27	32.93
Fundamental	27	29.35	19	23.17
Basic	14	15.22	12	14.63
High School	19	20.65	16	19.51
Higher education	2	2.17	8	9.76
Total	92	100.00	82	100.00

Socioeconomic status*				

A1 (RMF R$ 7793.00)	0	0.00	0	0.00
A2 (RMF R$ 4648.00)	0	0.00	3	3.75
B1 (RMF R$ 2804.00)	2	2.17	4	5.00
B2 (RMF R$ 1669.00)	9	9.78	8	10.00
C (RMF R$ 927.00)	58	63.04	43	53.75
D (RMF R$ 424.00)	22	23.91	22	27.50
E (RMF R$ 207.00)	1	1.09	0	0.00
Total	92	100.00	80	100.00

* Source: ABEP (2008)^14^; School *p* = 0.616; CSE: *p* = 0.054.

**Table 3:** Audiometric data and family past of hearing loss of the 174 patients, 92 from the SUS model and 82 from the Streamline model.

Hearing loss classifcation (best ear)*	SUS		Streamline	
	n	%	n	%
Normal	6	6.52	6	7.32
Mild	5	5.43	13	15.85
Moderate	22	23.91	21	25.61
Severe	44	47.83	30	36.59
Profound	15	16.30	12	14.63
Total	92	100.00	82	100.00

* Value of *p*: Mean value of the best ear thresholds: *p* = 0.079.

We did not find significant differences between the two groups as far as age and gender were concerned (Table 1) - enabling comparison between the two groups vis-à-vis the findings of the study. The largest group in both models was made up of women in the age range between 45 and 65 ears.

The same homogeneity profile was seen for schooling and socioeconomic status in the groups studied. Both in the Streamline as well as in the SUS groups, over 80% of the population belonged to C and D classes^13^ and without schooling or with complete fundamental education (over 50% in both groups) (Table 2), and there was no statistically significant difference in relation to these aspects.

Also, in relation to audiological characteristics found in both groups, there were no statistically significant differences, and most of the participants in both groups had mild hearing loss (Table 3).

As to the results from the IOI-HA questionnaire, for the SUS group, the mean scores for each question were between 4.34 and 4.87 and for the Streamline; the mean score of the participants was between 4.30 and 4.93 at three months (Table 4). During follow up at the ninth month, the mean score of the seven questions assessed varied between 4.28 and 4.85 for the SUS group and between 4.44 and 4.89 for the Streamline group (Table 5).

**Table 4:** Mean, standard deviation, median, minimum, maximum and percentiles 5 and 95% of the score of each question in the IOI questionnaire obtained for the groups assessed at three months.

IOI-HA																
	Use		Benefit		RAL		Satisfaction		RPR		IO		QL		Total	
	SUS	Streamline	SUS	Streamline	SUS	Streamline	SUS	Streamline	SUS	Streamline	SUS	Streamline	SUS	Streamline	SUS	Streamline
Minimum	1.00	1.00	3.00	2.00	3.00	2.00	3.00	4.00	2.00	2.00	2.00	3.00	3.00	3.00	26.00	22.00
Maximum	5.00	5.00	5.00	5.00	5.00	5.00	5.00	5.00	5.00	5.00	5.00	5.00	5.00	5.00	35.00	35.00
Mean	4.56	4.51	4.56	4.62	4.34	4.30	4.87	4.93	4.74	4.75	4.67	4.77	4.78	4.75	32.74	32.75
SD	0.94	0.92	0.52	0.56	0.60	0.62	0.36	0.24	0.55	0.53	0.63	0.45	0.46	0.45	2.17	2.17
Median	5.00	5.00	5.00	5.00	4.00	4.00	5.00	5.00	5.00	5.00	5.00	5.00	5.00	5.00	33.00	33.00
5%	2.00	3.00	4.00	4.00	3.00	3.00	4.00	4.00	4.00	4.00	4.00	4.00	4.00	4.00	29.00	28.00
95%	5.00	5.00	5.00	5.00	5.00	5.00	5.00	5.00	5.00	5.00	5.00	5.00	5.00	5.00	35.00	35.00

*P*	0.573		0.363		0.713		0.241		0.925		0.501		0.641		0.641	

RAL: Residual activity limitation; RPR: Residual participation restriction; IO: Impact on others; QL: Quality of life.

**Table 5:** Mean, standard deviation, median, minimum, maximum and percentiles 5% and 95% of the score from each question in the IOI questionnaire for the groups assessed at nine months.

IOI-HA																
	Use		Benefit		LRA		Satisfaction		RRP		IO		QV		Total	
	SUS	Streamline	SUS	Streamline	SUS	Streamline	SUS	Streamline	SUS	Streamline	SUS	Streamline	SUS	Streamline	SUS	Streamline
Minimum	1.00	1.00	4.00	3.00	2.00	2.00	4.00	4.00	1.00	3.00	1.00	3.00	3.00	4.00	24.00	25.00
Maximum	5.00	5.00	5.00	5.00	5.00	5.00	5.00	5.00	5.00	5.00	5.00	5.00	5.00	5.00	35.00	35.00
Mean	4.51	4.61	4.70	4.69	4.28	4.44	4.85	4.89	4.72	4.82	4.74	4.75	4.81	4.84	32.92	33.16
SD	1.09	0.80	0.45	0.49	0.67	0.65	0.35	0.30	0.63	0.41	0.66	0.48	0.42	0.36	1.93	1.96
Median	5.00	5.00	5.00	5.00	4.00	5.00	5.00	5.00	5.00	5.00	5.00	5.00	5.00	5.00	33.00	34.00
5%	1.00	3.00	4.00	4.00	3.00	3.00	4.00	4.00	4.00	4.00	4.00	4.00	4.00	4.00	30.00	29.00
95%	5.00	5.00	5.00	5.00	5.00	5.00	5.00	5.00	5.00	5.00	5.00	5.00	5.00	5.00	35.00	35.00

*P*	0.772		0.974		0.090		0.388		0.453		0.653		0.723		0.305	

RAL: Residual activity limitation; RPR: Residual participation restriction; IO: Impact on others; QL: Quality of life.

Insofar as the results from both groups are concerned, we did not find statistically significant differences in the total score from each question (in relation to use, benefit, satisfaction, residual limitation in activities, residual restriction in participation, impact on others and quality of life) and in the total score of the IOI-HA questionnaire at three (Table 4) and nine months (Table 5) after ISAD fitting.

What we see in the results is that, at 3 and 9 months, there was no variation between the two groups. At 3 months, both groups were equal in terms of the mean value of the Total in the IOI-HA questionnaire (means of 32.74 and 32.75, respectively). At 9 months we found a small difference (SUS = 32.92; Streamline = 33.16), however non-significant (*p* = 0.3051), in other words, we had no difference in using one or the other strategy in the total results from the questionnaire at three and nine months.

We found a statistically significant difference for the SUS group in the benefit question, in other words, after nine months of using ISAD there was an improvement vis-à-vis hearing difficulties with the hearing aid, and this was not found in the Streamline group (Table 6), also not found in relation to the total score; and the mean values from the two groups were very similar (Table 7).

**Table 6:** Statistical analysis (Wilcoxon test) to check whether there are differences between the scores from the two models investigated between 3 and 9 months.

	SUS	Streamline
	*p*	*p*
Use	0.794	0.515
Benefit	0.009*	0.227
Residual activity limitation	0.446	0.084
Satisfaction	0.445	0.311
Participation restriction	0.650	0.445
Impact on others	0.327	0.374
Quality of life	0.359	0.062
Total	0.231	0.071

* *p* value < 0.05 - statistically significant.

**Table 7:** Minimum, maximum, mean, median and standard deviation (SD) values of the Speech Recognition Index (SRI) obtained from groups SUS and Streamline before ftting, at three and nine months using the ISAD.

	SUS Group			Streamline Group		
	Before (Without ISAD)	3 m (With ISAD)	9 m (With ISAD)	Before (Without ISAD)	3 m (With ISAD)	9 m (With ISAD)
Minimum	0.00	0.00	0.00	0.00	0.00	0.00
Maximum	88.00	100.00	100.00	92.00	100.00	100.00
Mean	27.71	65.97	66.20	41.75	76.82	76.46
Median	0.00	80.00	80.00	54.00	90.00	90.00
SD	31.92	34.69	35.02	36.01	30.50	32.25

^*^p	-	0.6343		-	0.1897	

*p* value < 0.05 - statistically significant; SRI comparison of 3 and 9 months with the ISAD for both groups.

The Table 7 shows the results from the Speech Recognition Index (SRI) investigated in both groups before the fitting (without ISAD), three and nine months after fitting the hearing aid. In average, the Streamline group had a better Speech Recognition Index result before the fitting (without the ISAD) than in the SUS group. The SUS group went from 27.71% (before fitting - without ISAD) to 66.20% at 9 months and the Streamline group went from 41.71% (pre-fitting - without the ISAD) into 76.46% at 9 months with the ISAD. The difference for both groups after 9 months of fitting was, respectively 38.26% and 34.71%, and such finding was not significant (*p* = 0.6686).

## DISCUSSION

This study was done with the goal of comparing the two models of care: the SUS model, with a more compact and streamlined model of care vis-à-vis the individuals’ assessment of speech performance and important fitting domains, such as the use of ISAD, benefit, satisfaction, participation restriction, activities limitation, impact of the hearing loss on others and quality of life. The intent was to assess whether or not it would be possible to maximize care without losing quality.

Based on the results obtained, it was possible to notice that the two groups had the same improvement in relation to speech recognition after 9 months of hearing aid use. Although the two groups were homogeneous vis-à-vis the sociodemographical variables and audiological characteristics, we noticed a mild exit point difference in relation to speech perception in both groups, that is, the Streamline group had a better pre-fitting result without the hearing aid. This may have happened because the randomization process is not perfect for small groups and, moreover, the audiological profile of the patients was not taken into account in this group selection process. What we notice with this result is that the lower the speech recognition at the pre-fitting stage (without the ISAD), more improvement can be obtained during the 9 months, since the scale is shorter. Despite such difference, the speech performance results were similar for both groups at three and nine months after fitting the hearing aid.

Besides such findings, in the IOI-HA questionnaire results there were no statistically significant difference in the issues: use, benefit, satisfaction, residual limitation of activities, residual restriction of participation, impact on others and quality of life, and in the total score at three and nine months after fitting the ISAD, which shows that the type of care did not impact the results of this assessment.

The high scores in all these aspects assessed, vis-à-vis ISAD fitting (use, benefit, satisfaction, residual limitation in activities, participation restrictions, impact on others and quality of life) show that the participants from both groups had attitudes favoring their ISADs at three and nine months after fitting. The high mean values found were higher than those in the normative in English - similar results to the ones found in the studies carried out by Williams et al.^15^ and Gasparin et al.^16^. Other studies found mean values similar to the ones in the normative - such as the ones held by Cox & Alexander^17^ and Olusanya^18^. It is possible that, in Brazil, since the SUS pays for hearing aids, this may trigger a different attitude in the user in this type of assessment.

The statistically significant difference found in the SUS group was striking in the question associated with the benefit, in other words, after nine months using the ISAD there was an improvement vis-à-vis the difficulties in hearing with the hearing aid, and such fact was not found in the Streamline group. On the other hand, the care involving fewer procedures led to similar results as far as speech recognition is concerned for both groups, indicating that it must be better assessed in future studies.

This way, we could notice that the two care strategies did not differ from the clinical standpoint in regards of the results obtained from the patients concerning the ISAD fitting. This change in the routine of care, as per the protocol suggested for the auditory health services involving less procedures (only one ISAD test and with the physical placement of the equipment in only one room, avoiding fragmentation of care and of the instructions given to the patients in different rooms and by different professionals), did not impact the results obtained in the short and medium run vis-à-vis patient benefits, satisfaction and speech perception.

In this study, the anticipated choice of the NAL-NL1 method for the streamline group sped care and helped the audiologist be more focused on the job, providing good ISAD fitting results in this group. We must stress that the hearing health clinic audiologist must be prepared and be knowledgeable concerning the options provided in the ISAD adjustments, because even for validated methods, it is possible that the real gain is below the prescribed gain and the choice of a prescription rule must be based on scientific evidence and must be changed when the clinician finds that the patient's results are not as satisfying as expected.

Today, thanks to technological progress, almost all ISAD, of different brands and models, use technology which enables them to adapt to the specific needs of each patient^19^. While some ISAD manufacturers recommend validated procedures for prescribing the electroacoustic characteristics of the hearing aid, such as the prescriptive NAL-NL1 and DSL [i/o] methods, others have introduced their own algorithms for ISAD fitting^20^. What happens in clinical practice is that often times the validated prescription methods are not used or checked upon ISAD fitting in adults and, although such fact is not broadly documented, it is common for audiologist to program gain and output using the values prescribed by the manufacturers’ software and, in many a case, these algorithms may differ significantly from these methods^21^. It is also important to notice that the software algorithms are based on data pertaining to the mean value of scientific data values and always based on the 2cc coupler, which may not correspond to the patient at hand. It is important that the audiology clinic chose one prescriptive method with scientific evidence and acquire clinical experience with it so as to be able to setup its own protocol, accelerating decision making based on scientific research and on the experience of the clinic itself. This prevents the professionals from acting automatically without using clinical rationale in their own practice.

Another factor is that, today, with the technological progress of ISADs, it is possible to have different adjustments supplied in the same hearing aid device, depending on patient complaint during the tests with the devices. Each different setting programed can be checked by means of the probe microphone measures, which will assess the ISAD performance in an objective fashion, allowing for a better accuracy in the adjustments and on the assessment of the amplification received by the hearing impaired individual^22^. Such objective information, together with subjective patient information, will enable us to define which the best setup is at the fitting stage, not requiring other tests with ISAD of different brands, for later indication of a hearing aid. Nonetheless, there is still no scientific evidence supporting this issue, thus, all types of patients and hearing losses must be seen the same way, considering that this is an adult population.

Moreover, there are difficulties in obtaining three models of similar ISADs from different brands, so that the tests with patients may be carried out only after the device is indicated. Even three ISAD models from one and the same brand fail in this rigorous demand; thus, professional training and education is fundamental in this field. It is mandatory that the ISAD devices acquired by the SUS should keep a quality, enabling the necessary changes in adjustments and savings should be achieved by employing resources in the updating and motivation of the healthcare professionals to enable them to better fit ISADs, benefiting the population.

Another issue that deserves comments is the reduction in the time the health care professional spends with the patient, which is now of 45 minutes per fitting. This maximizes care and increases the number of patients seen. This was not the goal of the present investigation; therefore, its results were not discussed in this paper.

Since the clinical results attained were similar for both groups, in this study the Streamline group involved fewer professionals and audiological procedures in their routine care, and with these results we foresee the possibility of caring for a larger number of patients in the auditory healthcare services, and the changes hereby proposed must be considered in changes to Ordinance 587^10^.

After seven years of the National Policy for Auditory Health Care in our country, we have seen that it is paramount to control and nurture the processes for the population needing such care. The growing demand of patients seeking treatment for their hearing loss in recent years showed the need to think about the speed in seeing this population, having given that another model of care could speed up this process and enable us to see more patients, involving fewer professionals. We know that many auditory healthcare services today have repressed demands and proposals to tackle such problem are being discussed among different groups of professionals associated with scientific societies.

In comparison, in other countries there is a broad variety of auditory healthcare services provided and differences concerning the waiting time for a hearing aid. In Sweden, for instance, a person may wait 3 months to receive the device, and this time can be of 18 months in Finland. In some countries there are certain priority groups, for example, workers and children have priority and waiting time is shorter, as it happens in Denmark and Norway^23^. It is important to stress that we are proud to compare Brazil with other more developed societies in the world. This is possible because of our Public Healthcare System (SUS) and the National Policy for Auditory Healthcare.

Therefore, optimizing the flow of care will provide greater access of the population to treatment in due time and free up the healthcare teams to bring awareness about hearing preservation and other preventive actions - as proposed by the ordinance.

## CONCLUSION

This study enabled us to conclude that the two strategies of care did not differ vis-à-vis the clinical standpoint, nor concerning the results obtained in the assessment of patients for fitting hearing aids. The streamline model (only one ISAD test and with all the equipment placed in one single room), in regards of the assessment of those participating in the study concerning speech perception and important fitting domains - such as the use of an ISAD, benefits, patient satisfaction, participation restriction, activities limitation, hearing impairment impact on others and quality of life, did not impact on the results obtained in the short and medium runs, and all of this must be considered in changes to Ordinance 587^10^.
